# Occurrence and Potential Human-Health Relevance of Volatile Organic Compounds in Drinking Water from Domestic Wells in the United States

**DOI:** 10.1289/ehp.10253

**Published:** 2007-08-20

**Authors:** Barbara L. Rowe, Patricia L. Toccalino, Michael J. Moran, John S. Zogorski, Curtis V. Price

**Affiliations:** 1 U.S. Geological Survey, Rapid City, South Dakota, USA; 2 U.S. Geological Survey, Sacramento, California, USA

**Keywords:** domestic wells, drinking-water quality, human health, volatile organic compounds

## Abstract

**Background:**

As the population and demand for safe drinking water from domestic wells increase, it is important to examine water quality and contaminant occurrence. A national assessment in 2006 by the U.S. Geological Survey reported findings for 55 volatile organic compounds (VOCs) based on 2,401 domestic wells sampled during 1985–2002.

**Objectives:**

We examined the occurrence of individual and multiple VOCs and assessed the potential human-health relevance of VOC concentrations. We also identified hydrogeologic and anthropogenic variables that influence the probability of VOC occurrence.

**Methods:**

The domestic well samples were collected at the wellhead before treatment of water and analyzed for 55 VOCs. Results were used to examine VOC occurrence and identify associations of multiple explanatory variables using logistic regression analyses. We used a screening-level assessment to compare VOC concentrations to U.S. Environmental Protection Agency maximum contaminant levels (MCLs) and health-based screening levels.

**Results:**

We detected VOCs in 65% of the samples; about one-half of these samples contained VOC mixtures. Frequently detected VOCs included chloroform, toluene, 1,2,4-trimethylbenzene, and perchloroethene. VOC concentrations generally were < 1 μg/L. One or more VOC concentrations were greater than MCLs in 1.2% of samples, including dibromochloropropane, 1,2-dichloropropane, and ethylene dibromide (fumigants); perchloroethene and trichloroethene (solvents); and 1,1-dichloroethene (organic synthesis compound).

**Conclusions:**

Drinking water supplied by domestic wells is vulnerable to low-level VOC contamination. About 1% of samples had concentrations of potential human-health concern. Identifying factors associated with VOC occurrence may aid in understanding the sources, transport, and fate of VOCs in groundwater.

Groundwater is used as a drinking-water supply by about one-half of the U.S. population, including almost all people residing in rural areas. As estimated by the U.S. Geological Survey (USGS) (Hutson et al. 2004), domestic wells provide drinking water to about 43.5 million people, representing 15% of the total U.S. population (Supplemental Material, Figure 1, available online at http://www.ehponline.org/members/2007/10253/suppl.pdf). Estimated withdrawals from domestic wells increased by 60% between 1965 and 2000, with an average withdrawal rate of about 3.6 billion gallons per day in 2000 (Hutson et al. 2004). Between 1995 and 2000, domestic withdrawals increased about 6%, and domestic population increased almost 2% (Hutson et al. 2004), indicating increased use of self-supplied drinking water. In addition, estimates by the National Ground Water Association indicate that > 400,000 new domestic wells used for drinking-water supplies are drilled each year in the United States (McCray KB, personal communication).

As the population and demand for safe drinking water from domestic wells increase, it is important to examine water quality and identify contaminants that occur in water from domestic wells. One contaminant group of concern is volatile organic compounds (VOCs), which are contained in many products used around households, including solvents, paints, adhesives, deodorizers, refrigerants, fuels, and fumigants. A VOC is an organic chemical that has a high vapor pressure relative to its water solubility. The chemical and physical properties of VOCs allow the compounds to move between the atmosphere, soil, surface water, and groundwater. Once in the environment, VOCs can be mobilized, dispersed, diluted, volatilized, adsorbed, and/or degraded. Although many VOCs have relatively short half-lives in certain media due to abiotic and biotic degradation, other VOCs can be persistent, degrading little over years or decades. The production of some synthetic organic chemicals (many of which are VOCs) has increased by more than an order of magnitude between 1945 and 1985 (Ashford and Miller 1991). Some VOCs, such as chlorinated solvents, have been used in industry and commerce for almost 100 years (Pankow and Cherry 1996). Once introduced to groundwater, VOCs may persist and potentially contaminate drinking-water supplies.

The U.S. Environmental Protection Agency (EPA) reported that the presence of elevated VOC concentrations in drinking water may be a concern to human health because some VOCs are carcinogens and/or may adversely affect the liver, kidneys, spleen, and stomach, as well as the nervous, circulatory, reproductive, immune, cardiovascular, and respiratory systems (U.S. EPA 2003, 2007a). Some VOCs may affect cognitive abilities, balance, and coordination, and some are eye, skin, and/or throat irritants. The Agency for Toxic Substances and Disease Registry (ATSDR 2007) provides human-health information that is searchable by individual contaminant, and additional information is provided by the U.S. EPA (2003, 2007a, 2007b).

The USGS’s National Water-Quality Assessment (NAWQA) Program recently completed an assessment of 55 VOCs in groundwater throughout the United States. A screening-level assessment used in that study compared VOC concentrations to human-health benchmarks for drinking water to aid in understanding the potential human-health relevance of VOC occurrence (Zogorski et al. 2006). VOC concentrations in samples collected before treatment or blending during 1985–2002 from 2,401 domestic wells were compared with human-health benchmarks when available, including U.S. EPA maximum contaminant levels (MCLs) for regulated contaminants and health-based screening levels (HBSLs) for unregulated contaminants (those without U.S. EPA MCLs) (Toccalino et al. 2003). HBSLs, as well as MCLs, are maximum contaminant concentrations that are not expected to cause adverse health effects over a lifetime of exposure (Toccalino 2007).

MCLs, established under provisions of the Safe Drinking Water Act, are legally enforceable U.S. EPA drinking-water standards (U.S. EPA 2006e) that set the maximum permissible level of a contaminant in water that is delivered to any user of a public water system. MCLs are set as close as feasible to the maximum level of a contaminant at which no known or anticipated adverse effects on human health would occur over a lifetime, taking into account the best available technology, treatment techniques, cost considerations, expert judgment, and public comments (U.S. EPA 2006f).

HBSLs are not legally enforceable drinking-water standards or clean-up levels. Rather, HBSLs are nonenforceable benchmark concentrations in water that, when exceeded, may be of potential human-health concern. HBSLs were developed by the USGS in collaboration with the U.S. EPA, the New Jersey Department of Environmental Protection (Trenton, NJ), and the Oregon Health & Science University (Portland, OR) using standard U.S. EPA Office of Water equations for establishing drinking-water guideline values (lifetime health advisory and cancer risk concentration values) for the protection of human health, and the most current U.S. EPA peer-reviewed, publicly available human-health toxicity information (Toccalino et al. 2003). HBSLs are based on health effects and do not consider cost or technical limitations (Toccalino 2007; Toccalino and Norman 2006; Toccalino et al. 2003) (Supplemental Material, Table 1, available online at http://www.ehponline.org/members/2007/10253/suppl.pdf).

Drinking water from domestic wells is not regulated by federal standards and typically does not receive the same level of monitoring and treatment as drinking water supplied by public water systems (U.S. EPA 2003). Although regulations vary by state, the quality of water from privately owned domestic wells is the homeowner’s responsibility.

This national assessment of 55 VOCs in drinking water from 2,401 domestic well samples has three primary objectives: *a*) to examine the occurrence of individual VOCs and VOC mixtures; *b*) to assess the potential human-health relevance of individual VOCs that have established human-health benchmarks; and *c*) to link VOC occurrence to hydrogeologic and anthropogenic variables that potentially control or influence the occurrence of VOCs in groundwater.

## Methods

### VOC selection

We selected the VOCs in this study on the basis of available information and the feasibility of laboratory analysis of VOCs by purge and trap gas chromatography/mass spectrometry (P&T GC/MS) (Bender et al. 1999). Equipment used for P&T GC/MS included the Tekmar 3000 Concentrator and the Tekmar Velocity Concentrator (Teledyne Tekmar, Mason, OH) and the Agilent 6890 GC and Agilent 5973 MSD (Agilent Technologies Inc., Santa Clara, CA). Selection of candidate VOCs involved many criteria, including potential human-health cancer risks and noncancer hazards, toxicity to and bioconcentration in freshwater aquatic organisms, physical properties and occurrence statistics, use or potential use as an oxygenate in gasoline, and potential for atmospheric ozone depletion (Bender et al. 1999). Although many VOCs have multiple uses, each compound was placed in a group representing the predominant use (or origin) of the compound; these include fumigants, gasoline hydrocarbons, gasoline oxygenates, organic synthesis compounds (VOCs used in the formation of other organic compounds), refrigerants, solvents, and trihalomethanes (THMs, disinfection by-products).

### NAWQA data

Domestic well data include 2,401 samples collected during 1985–2002 and represent > 33 of the nation’s 62 regionally extensive aquifers or aquifer systems (Zogorski et al. 2006). The depth of sampled wells ranged from 6 to 1,500 ft and had a median depth of about 140 ft. The term “sample” represents a distinct geographic site and applies to an environmental domestic well sample collected at the wellhead before household treatment. The characterization of water quality was achieved by sampling 20–30 spatially distributed, randomly selected wells throughout network-based, groundwater studies (Gilliom et al. 1995) including *a*) large areal and depth dimensions of aquifers considered locally and regionally important (1,621 samples); and *b*) shallow, recently recharged groundwater samples (247 samples from agricultural land-use areas and 16 samples from urban land-use areas).

To supplement NAWQA VOC data, we compiled existing domestic well data collected by federal, state, and local agencies (retrospective data). Retrospective data that met NAWQA design characteristics and data-collection procedures (Lapham and Tadayon 1996) provided information for an additional 517 domestic wells sampled during 1985–1995.

### Sampling and analytical methods

A single sample collected at each well represents the water quality. Samples were collected at the wellhead before any treatment or holding time in tanks. Most samples were collected by USGS personnel using data-collection protocol and quality-control procedures (Koterba et al. 1995) and analyzed at the USGS National Water Quality Laboratory using P&T GC/MS. Analytical methods and quality-control samples used in this study have been reported elsewhere (Childress et al. 1999; Connor et al. 1998; Rose and Schroeder 1995).

Before April 1996, USGS VOC analytical methodology was similar to U.S. EPA method 524.2, revision 3 (U.S. EPA 1995), using a minimum reporting level (MRL) of 0.2 μg/L. This MRL represents the occurrence of VOCs at a historical reporting value for the USGS and other agencies. An enhanced method for the VOC analysis, implemented in April 1996 by the USGS National Water Quality Laboratory, allowed the reporting of VOC concentrations less than the historical MRLs (Connor et al. 1998). Application of this USGS low-level analytical method to domestic well samples resulted in substantially lower reporting levels for many VOCs. Laboratory reporting levels for most VOCs were different from one another, and these levels also varied as method changes were implemented or new instrumentation was used (Supplemental Material, Table 2, available online at http://www.ehponline.org/members/2007/10253/suppl.pdf).

### Reporting of VOC data

The VOC data set was examined in two ways to address the objectives of this study. Analytical results from the total 2,401 domestic wells sampled (1985–2002), including 1,193 samples analyzed at an MRL of 0.2 μg/L and 1,208 samples analyzed with the low-level method, were used to gain a broad perspective on VOC concentrations relative to human-health benchmarks. Using data from all 2,401 domestic well samples maximized the number of VOC concentrations that could be compared to human-health benchmarks, thereby providing a basis for a more comprehensive evaluation of VOC occurrence data in the context of human health.

We report VOC occurrence findings, including detection frequencies, concentrations, and spatial distributions, using data from a subset of the samples—that is the 1,208 samples collected and analyzed (1996–2002) with the low-level method. These data provide a relevant assessment of low-level VOC concentrations that are present in ambient groundwater (untreated water characteristic of the aquifer resources) and best describe VOC concentrations that may be present in drinking water supplied by domestic wells. The low-level analytical method also provides the highest analytical resolution data for multivariate statistical analyses of individual compounds to determine explanatory factors associated with VOC occurrence. Furthermore, as precision of analytical methodologies improves, the USGS low-level VOC data in this study can effectively be used in future comparative analyses for assessment of long-term VOC occurrence trends. Based on these collective objectives, low-level analytical results for VOC occurrence from 1,208 samples are reported with no censoring of data.

### Statistical analyses

We used non-parametric statistical tests to analyze VOC occurrence data. Multivariate logistic regression analyses were used to determine relations between probability of VOC occurrence relative to hydrogeologic and anthropogenic variables. Information on this statistical approach and variables (Moran et al. 2006) is available in Appendix 1 of the Supplemental Material (available online at http://www.ehponline.org/members/2007/10253/suppl.pdf). The significance of logistic regression analyses was tested using various statistical criteria (Helsel and Hirsch 1992). We computed standardized coefficients to compare slope coefficients directly between one another (Menard 2002).

### Screening-level assessment

Of the 42 VOCs detected in domestic well samples, 35 have established human-health benchmarks. VOC concentrations for 27 regulated compounds were compared to their MCLs, and concentrations for 8 unregulated VOCs were compared to their HBSLs. VOC concentrations of potential human-health concern in domestic well samples were defined as those concentrations greater than MCLs or HBSLs. We identified VOC concentrations within one order of magnitude of MCLs or HBSLs as compounds that may warrant additional monitoring to analyze trends in occurrence and to provide an early indication of concentrations approaching human-health benchmarks (Toccalino and Norman 2006). State and federal agencies use a variety of thresholds (typically one-tenth or one-half of a human-health benchmark) to identify contaminants that may warrant additional monitoring (U.S. EPA 2000, 2002) or for related purposes, such as ranking the susceptibility of wells to contamination (New Jersey Department of Environmental Protection 2003, 2004) and identifying contaminants of potential human-health concern (U.S. EPA 1993). Using concentrations within one order of magnitude of a human-health benchmark to identify compounds that may warrant additional monitoring is therefore consistent with various state and federal practices.

## Results

### VOC occurrence

We detected one or more VOCs in 65% of the domestic well samples. Of the 55 VOCs monitored, 42 compounds were detected. Detection frequencies were > 10% for chloroform (25.6%), toluene (17.9%), 1,2,4-trimethylbenzene (1,2,4-TMB; 15.2%), and perchloroethene (PCE; 11%) (Table 1). Nineteen VOCs had detection frequencies between 1 and 10%, and 18 VOCs had detection frequencies > 0.1 and < 1% (Table 1). The 15 frequently detected compounds represent all VOC groups except fumigants. These VOCs have widespread applications and multiple uses, and most compounds were detected throughout the conterminous United States and in Alaska. VOC concentrations generally were low; of the sampled wells, 91% had total VOC concentrations ≤1 μg/L and about 1% had total VOC concentrations > 10 μg/L.

Of the well samples, 31% had a single VOC detection and 34% had VOC mixtures, which we defined as two or more VOCs that are present in a domestic well sample. The 10 most frequently detected VOCs each occurred more frequently in samples with mixtures than as single VOCs in samples (Supplemental Material, Table 3, available online at http://www.ehponline.org/members/2007/10253/suppl.pdf). The median concentrations for these VOCs generally were greater when detected in a mixture than alone. Of the 10 frequently detected VOCs, methyl *tert*-butyl ether (MTBE) had the greatest median concentration both alone and in mixtures. Median concentrations generally were greatest for gasoline oxygenates and refrigerants, and median concentrations were lowest for fumigants and gasoline hydrocarbons.

We detected as many as 24 individual VOCs in one sample; however, the most frequently occurring mixtures were composed of two or three unique compounds. Chloroform was a common constituent in the four most frequently detected mixtures, co-occurring in two-compound mixtures with, in decreasing order of detection frequency, 1,1,1-trichloroethane (1,1,1-TCA), PCE, toluene, and MTBE (Supplemental Material, Table 4, available online at http://www.ehponline.org/members/2007/10253/suppl.pdf). The co-occurrence of chloroform with a VOC from another group (e.g., PCE) may result from wide distribution of these compounds that spatially overlap. VOC mixtures, such as PCE and 1,1,1-TCA, may co-occur because the physical and chemical properties of these compounds could result in similar environmental behavior. Mixtures may also result from VOCs sharing the same source, such as toluene and 1,2,4-TMB in gasoline products. Mixtures such as PCE and trichloroethene (TCE) may result from the degradation of the parent compound (PCE) to the by-product (TCE).

### Relational analyses

Results of logistic regression analyses for frequently detected VOCs and associated variables are summarized in Table 2. VOCs that had a variable with a standardized coefficient ≥0 indicate that as the variable increases, the probability of detecting that compound increases; conversely, VOCs that had a variable with a standardized coefficient < 0 indicate that as the variable increases, the probability of detecting that compound decreases. If a standardized coefficient of a variable for a VOC was ≥0.1 (absolute value), the variable was considered to be strongly associated with the probability of a compound’s occurrence; if a standardized coefficient of a variable for a VOC was < 0.1 (absolute value), the variable was considered to be weakly associated with the probability of the compound’s occurrence. Based on the strength of variable associations and the frequency of VOC detections, the five variables in order of decreasing importance were *a*) dissolved-oxygen content; *b*) precipitation; *c*) the number of Resource Conservation and Recovery Act [RCRA (U.S. EPA 2006c)] sites (that generate, transport, store, or dispose of hazardous waste materials) within a 1-km radius of the well; *d*) aquifer type; and *e*) water temperature.

The hydrogeologic variable we found to be most strongly and frequently associated with VOC occurrence in domestic well water was dissolved-oxygen content, which is a key factor in biodegradation. The probability of detecting chloroform, MTBE, 1,1,1-TCA, and toluene increased with increasing dissolved-oxygen content (Table 2), whereas the probability of detecting chloromethane decreased with increasing dissolved-oxygen content. The probability of detecting MTBE increased with increasing precipitation, as expected. Precipitation is the driving force for recharge and the transport of VOCs from land surface to the water table. The probability of detecting MTBE decreased with increasing depth to the top of the well’s screened interval. Depth to the top of the screened interval is believed to be roughly equivalent to the depth to the top of the aquifer (Moran et al. 2006). Increased depth to the screened interval allows more travel time from the MTBE source to the aquifer, which may allow for increased attenuation of MTBE concentrations through natural loss processes such as biodegradation, sorption, dispersion, and volatilization. Furthermore, the probability of detecting MTBE decreased with increasing water temperature, which is believed to be related to the biologic activity necessary for transformation of the compound (Moran et al. 2006).

The anthropogenic variable most strongly and frequently associated with probability of VOC occurrence was the number of RCRA sites within a 1-km radius of the well. The probability of detecting three solvents—PCE, TCE, and 1,1,1-TCA—increased with increasing number of sites near the wells. The probability of detecting the oxygenate MTBE increased with increasing number of leaking underground storage tank sites within a 1-km radius of the well.

### Comparison of VOC concentrations to human-health benchmarks

**Figure 1:**
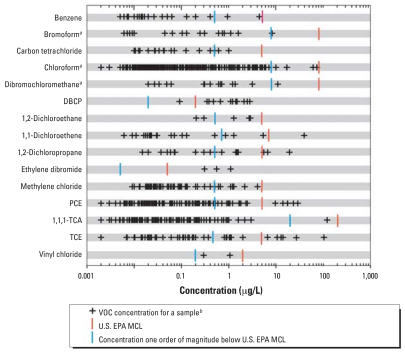
Distribution of 15 VOCs with concentrations greater than and/or within one order of magnitude of U.S. EPA MCLs in 2,401 domestic well samples. ^***a***^The U.S. EPA MCL of 80 μg/L is the sum of the concentration of four THMs. ^***b***^Multiple samples with equal concentrations appear as a single symbol.

One or more VOC concentrations were greater than a human-health benchmark in 1.2% of the domestic well samples. Six VOCs had concentrations greater than MCLs: dibromochloropropane (DBCP), 1,2-dichloropropane, ethylene dibromide (fumigants); PCE and TCE (solvents); and 1,1-dichloroethene (organic synthesis compound) (Table 3 and Figure 1). With the exception of ethylene dibromide, these VOCs also had concentrations less than but within one order of magnitude of their MCL in samples. One or more VOCs were detected within one order of magnitude of their MCL in 2.1% of the samples and include nine regulated VOCs: benzene (gasoline hydrocarbon); vinyl chloride (organic synthesis compound); carbon tetrachloride, 1,2-dichloroethane, methylene chloride, and 1,1,1-TCA (solvents); and bromoform, chloroform, and dibromochloromethane (THMs) [Figure 1 and Supplemental Material, Table 5 (available online at http://www.ehponline.org/members/2007/10253/suppl.pdf)]. No VOC concentrations were greater than or within one order of magnitude of HBSLs. Seven VOCs detected in samples but without established MCLs or HBSLs were, in order of decreasing detection frequency, MTBE, 1,1-dichloroethane, 1,2,4-TMB, chloroethane, *tert*-amyl methyl ether, diisopropyl ether, and *n*-propylbenzene.

VOCs with concentrations of potential human-health concern commonly occurred in mixtures. Of the 32 VOC concentrations greater than MCLs, 23 were in samples containing VOC mixtures. Of the 67 VOC concentrations within one order of magnitude of MCLs, 37 were in samples containing VOC mixtures.

**Figure 2:**
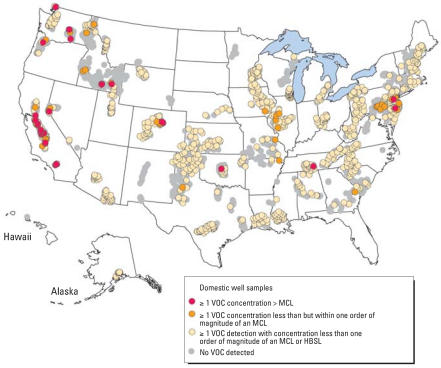
Locations of domestic wells sampled for VOCs and locations of measured VOC concentrations relative to human-health benchmarks, including U.S. EPA MCLs for regulated VOCs and to HBSLs for unregulated VOCs.

Sampling locations and spatial distributions of VOC concentrations in the United States relative to human-health benchmarks are shown in Figure 2. Samples with VOC concentrations greater than MCLs generally were localized within regions or states (Supplemental Material, Table 6, available online at http://www.ehponline.org/members/2007/10253/suppl.pdf) and generally were detected in highly populated areas, such as New Jersey, or were potentially associated with a particular VOC use, such as the historical application of DBCP on crops in the Central Valley of California (Burow et al. 1999). Samples with ≥1 VOC concentration within one order of magnitude of an MCL were distributed throughout the conterminous United States, and samples with VOC concentrations less than one order of an MCL or HBSL were distributed throughout the conterminous United States and in Alaska (Figure 2).

We also examined the 15 VOCs that had concentrations greater than and/or within one order of magnitude of MCLs in the 2,401 domestic well samples relative to their overall detection frequencies. The solvents methylene chloride, PCE, and TCE were among the most frequently detected compounds, and compared with other VOCs, had a large percentage of samples with concentrations greater than or within one order of magnitude of an MCL (Supplemental Material, Figure 2, available online at http://www.ehponline.org/members/2007/10253/suppl.pdf). These solvents are important VOCs to consider for source control and monitoring programs at local, state, and national levels.

## Discussion

### Scope of NAWQA study and relevance of findings

Several groundwater studies have focused on select VOCs or VOC groups. A study by the State of Maine (1998) focused on MTBE, benzene, toluene, ethylbenzene, and xylene (State of Maine 1998). Since the 1980s the Wisconsin Department of Natural Resources (2003) has tested for VOCs in water from domestic wells that were known or suspected to be vulnerable to VOC contamination. In a NAWQA study, Moran et al. (2002) reported preliminary VOC occurrence information on 55 VOCs based on 1,926 domestic well samples using a reporting level of 0.2 μg/L. The present study is the first national investigation to report the occurrence of a large number of VOCs in ambient groundwater from domestic well samples analyzed using low-level analytical methods and to assess the potential human-health relevance of individual VOC concentrations that have established human-health benchmarks.

About two-thirds of the NAWQA wells sampled throughout the United States since 1996 contained low-level VOC concentrations, indicating that VOC contamination of drinking water supplied by domestic wells may be more prevalent than previously reported by monitoring programs that used analytical methods with higher reporting levels, such as those described by Squillace et al. (1999) and the State of Maine (1998). As analytical reporting levels decrease, the detection frequency of VOCs increases; however, this does not necessarily indicate differences in water quality from a human-health perspective, but may reflect the greater resolving power of analytical instruments.

Mixtures were a common mode of VOC occurrence in domestic well samples; however, chemical regulation and toxicologic research have historically focused on individual chemicals rather than on mixtures (Monosson 2005). Furthermore, few data are available on the potential human-health effects resulting from long-term exposure to multiple compounds at low concentrations. The present study identified frequently detected VOC mixtures, and in addition, a NAWQA study now in progress is assessing multicontaminant occurrence of nitrate, radon, pesticides, VOCs, and trace elements (including arsenic and uranium) from domestic well samples throughout the United States. Identification of commonly occurring mixtures may aid the U.S. EPA and others in prioritizing future toxicologic and risk-assessment research.

Results of the relational analyses of VOCs in samples with variables reinforce the importance of identifying VOC sources and assessing the vulnerability of drinking-water supplies to VOCs. Identification of hydrogeologic and anthropogenic variables associated with the occurrence of an individual VOC, when coupled with the knowledge of the behavior and fate of the compound, can aid in understanding the vulnerability of aquifers to VOC contamination.

We found VOC concentrations to be generally lower than human-health benchmarks; concentrations of six VOCs in 1.2% of the samples were of potential human-health concern because their concentrations were greater than MCLs. With the exception of 1,1-dichloroethene, all of these compounds are regulated as carcinogens in drinking water (U.S. EPA 2006b). The potential health effects of these six VOCs with concentrations greater than MCLs are summarized in Table 3. Ingestion of water containing VOC concentrations greater than MCLs or HBSLs does not necessarily indicate that adverse human-health effects will occur. Human-health benchmarks are conservative (protective) because they incorporate safety factors to account for uncertainty in toxicity information and are based on lifetime exposure. Additionally, all samples in this study were collected at the wellhead before treatment. Treatment of water from domestic wells may reduce VOC concentrations at the tap (Toccalino et al. 2006).

Nine additional VOCs had concentrations less than but within one order of magnitude of MCLs (Supplemental Material, Table 5, available online at http://www.ehponline.org/members/2007/10253/suppl.pdf). These findings indicate that 15 VOCs may warrant inclusion in regional and national low-concentration, trends-monitoring programs. The potential human-health relevance for the 7 VOCs that were detected in samples but do not have established MCLs or HBSLs cannot be evaluated at this time. However, 1,2,4-TMB, MTBE, and 1,1,-DCA are presently included on the U.S. EPA’s Contaminant Candidate List and are prioritized for monitoring and data collection for public water systems (U.S. EPA 2006a).

### Potential human exposure to VOCs by ingestion of drinking water from domestic wells

The effective protection and management of the quality of drinking water supplied by domestic wells are shared responsibilities among well owners, governmental agencies, research organizations, and academic institutions. Proactive domestic well owners can properly maintain wells, routinely test drinking water, and implement management practices around the home to reduce VOC contamination. Although drinking water from domestic wells is not regulated by federal standards, some homeowners are improving their drinking-water quality by using granular activated-carbon filtering systems and filters mounted on taps and refrigerator dispensers. Reverse osmosis also is used to treat drinking water. Domestic well owners may rely on water supplied by jugs with filters or on bottled water for consumption. At the federal level, bottled water is regulated as a packaged food product and is governed by the U.S. Food and Drug Administration by Standards of Quality (U.S. EPA 2004).

A survey of 373 households was conducted by Probe Research Inc. (2005) to characterize drinking water consumed by domestic well users in the United States. An estimated 52% of the surveyed households indicated that drinking water was used straight from the tap with no treatment. If the statistics from the Probe Research Inc. drinking-water survey are representative of drinking water ingested by members of households throughout the United States, > 22 million people are drinking untreated domestic well water straight from the tap. Furthermore, 65% of the domestic well samples analyzed with NAWQA’s low-level method contained one or more VOCs. Applying this VOC detection frequency to the statistics provided by Probe Research Inc. (2005) indicates that > 14 million people throughout the conterminous United States and Alaska may be exposed to low VOC concentrations in their drinking water. In addition, 1.2% of the domestic wells sampled in the NAWQA assessment contained at least one VOC concentration greater than its MCL, indicating that as many as 300,000 domestic well users may be potentially ingesting drinking water that contains one or more VOCs at concentrations greater than MCLs.

The number of domestic well users exposed to VOCs may be underestimated using data from Probe Research Inc. (2005). Calculations based on the Probe Research Inc. (2005) survey indicate that nearly 9 million additional domestic well users rely on household-water treatment systems for removal of VOCs from drinking water; however, the effectiveness of VOC removal by in-house water-treatment systems is undetermined. Evaluating the effective removal of VOCs by in-house treatment methods from water supplied by domestic wells would necessitate routine monitoring, which presently is not required.

Most states and some local agencies provide guidance to well owners through web sites and printed materials. Guidance varies by state; however, information provided may include guidelines for well construction, proper well maintenance, preventative measures for contaminants, recommendations for water-quality testing, and certified treatment devices (U.S. EPA 2006d). Some states have initiated measures to assess water quality to aid in the protection of human health. For example, New Jersey passed a law in 2002 that requires private domestic well owners to test “raw” or untreated water for contaminants, including regulated VOCs, and to disclose results before selling or leasing properties (New Jersey Department of Environmental Protection 2006). Furthermore, some lawmakers have introduced bills in Congress that would mandate testing of private wells throughout the United States.

### Future direction and challenges

Science-based strategies are needed for identification of contaminants that are not regulated but may be present in drinking water from domestic wells. In addition, assessing the occurrence of contaminants from products that may be of current and future concern (e.g., personal care products, food additives, detergents, and pharmaceuticals) is warranted. Providing information on the occurrence of contaminants in drinking water from domestic wells is important for local, state, and federal water-resource managers and others charged with protecting and managing drinking-water resources. In addition, because many organic contaminants do not have human-health benchmarks, continued research is needed to provide the toxicologic data necessary to develop these benchmarks, which in turn provide tools for evaluating water-quality data in the context of human health.

Previously sampled wells, such as those included in the present study, could be resampled to further assess water-quality conditions and to determine variability and trends in contaminant occurrence and concentrations. Data collected systematically over a period of time could aid in determining whether a correlation exists between an identified contaminant source and VOC occurrence in a drinking-water supply. In addition, domestic wells that are located near areas that are known to be associated with contaminant sources could be targeted for monitoring.

At domestic well sampling sites, well characteristics and pumping information may be available; however, a challenge for future studies is to obtain ancillary information relevant to human health for domestic well users. For example, standard questionnaires could accompany sampling activities. If used, the type of household water-treatment systems could be identified, such as granulated activated-carbon for removal of VOCs, and supplementary drinking-water sources, including bottled water, could be determined. Additional pertinent information could include the number and age of household members, amount of water ingested, length of time using the water supply, and upgrades or changes in the household-water system since the initial installation at the time of the home construction.

As population, urbanization, and the demand for drinking water from domestic wells increase, continued evaluation of water quality is important. Research, including design, analysis, compilation of data, and comparison of measured contaminant concentrations to human-health benchmarks, can be used to assess the drinking-water quality from domestic wells and to prioritize investigations in a systematic effort at and among local, state, and national levels.

## Figures and Tables

**Table 1:** Detection frequencies of volatile organic compounds (VOCs)^a^ in 1,208 domestic well samples analyzed with the USGS’s low-level analytical method and reported with no censoring of data.

Compound	VOC group	No. of well samples	No. of detections	Detection frequency (%)
Chloroform	THM	1,207	309	25.6
Toluene	Gasoline hydrocarbon	1,203	215	17.9
1,2,4-TMB	Gasoline hydrocarbon	1,190	181	15.2
PCE	Solvent	1,179	130	11.0
Chloromethane	Solvent	1,207	117	9.7
1,1,1-TCA	Solvent	1,208	103	8.5
Methylene chloride	Solvent	1,207	74	6.1
MTBE	Gasoline oxygenate	1,208	67	5.5
Dichlorodifluoromethane	Refrigerant	1,208	43	3.6
TCE	Solvent	1,207	41	3.4
Benzene	Gasoline hydrocarbon	1,208	37	3.1
Bromodichloromethane	THM	1,207	34	2.8
*m-* and *p*-Xylene^b^	Gasoline hydrocarbon	1,206	28	2.3
1,1-Dichloroethane	Solvent	1,207	27	2.2
Styrene	Gasoline hydrocarbon	1,202	26	2.2
1,4-Dichlorobenzene	Fumigant	1,208	23	1.9
Trichlorofluoromethane	Refrigerant	1,208	23	1.9
Bromoform	THM	1,206	22	1.8
1,1-Dichloroethene	Org syn	1,207	19	1.6
Chlorobenzene	Solvent	1,208	16	1.3
Carbon tetrachloride	Solvent	1,207	13	1.1
Dibromochloromethane	THM	1,207	13	1.1
*cis*-1,2-Dichloroethene	Solvent	1,207	11	0.91
1,2-Dichloropropane	Fumigant	1,207	9	0.75
Isopropylbenzene	Gasoline hydrocarbon	1,208	9	0.75
*o*-Xylene	Gasoline hydrocarbon	1,205	8	0.66
Ethylbenzene	Gasoline hydrocarbon	1,208	7	0.58
*tert*-Amyl methyl ether	Gasoline oxygenate	1,206	6	0.50
1,3-Dichlorobenzene	Solvent	1,208	6	0.50
Trichlorotrifluoroethane	Refrigerant	1,207	6	0.50
Chloroethane	Solvent	1,207	4	0.33
1,2-Dichlorobenzene	Solvent	1,208	4	0.33
Diisopropyl ether	Gasoline oxygenate	1,096	4	0.36
DBCP	Fumigant	1,208	3	0.25
Naphthalene	Gasoline hydrocarbon	1,208	3	0.25
*n*-Propylbenzene	Solvent	1,208	3	0.25
*n*-Butylbenzene	Gasoline hydrocarbon	1,208	2	0.17
1,2-Dichloroethane	Solvent	1,208	2	0.17
1,2,3-Trichloropropane	Fumigant	1,208	2	0.17
1,1,2-Trichloroethane	Solvent	1,208	1	0.083
Vinyl chloride	Org syn	1,208	1	0.083

Abbreviations: DBCP, dibromochloropropane; MTBE, methyl *tert*-butyl ether; Org syn, organic synthesis compound; PCE, perchloroethene; 1,1,1-TCA, 1,1,1-trichloroethane; TCE, trichloroethene; TMB, 1,2,4-trimethylbenzene. VOCs are listed in order of decreasing detection frequency.

a VOCs not detected: acrolein, acrylonitrile, bromomethane, *trans*-1,2-dichloroethene, *cis*-1,3-dichloropropene, *trans*-1,3-dichloropropene, ethyl *tert-*butyl ether, ethylene dibromide, hexachlorobutadiene, hexachloroethane, 1,2,3-trichlorobenzene, 1,2,4-trichlorobenzene, and vinyl bromide.

b Considered as 2 of the 55 compounds included in this assessment.

**Table 2:** Alphabetized listing of frequently detected VOCs and statistical summary of hydrogeologic and anthropogenic variables associated with the probability of occurrence of the compound in domestic well samples.^a^

Compound	No. of samples in analyses	Associated variables^b^	Type of variable	Coefficient in logistic regression equation	Standardized coeffiicient
Chloroform	842	Dissolved-oxygen content	Fate	1.142	0.20
		Depth to water level	Transport	−0.005	−0.18
Chloromethane	1,182	Dissolved-oxygen content	Fate	−0.990	−0.15
		Recharge	Transport	−0.002	−0.07
Dichlorodifluoromethane	1,184	Water temperature	Fate	−0.087	−0.06
Methylene chloride	1,168	Dissolved-oxygen content	Fate	−0.536	−0.08
		Depth of well	Transport	−0.003	−0.13
		Permeability of soil	Transport	0.104	0.09
MTBE	969	Aquifer type	Transport	−0.943	−0.07
		Dissolved-oxygen content	Fate	1.291	0.10
		Depth to top of screened interval	Transport	−0.008	−0.15
		LUST sites within 1 km of well^c^	Source	1.907	0.10
		Precipitation	Transport	0.073	0.17
		Water temperature	Fate	−0.184	−0.13
PCE	1,155	Dissolved-oxygen content	Fate	0.384	0.09
		RCRA sites within 1 km of well^c^	Source	0.158	0.16
1,1,1-TCA	1,184	Dissolved-oxygen content	Fate	2.592	0.30
		RCRA sites within 1 km of well^c^	Source	0.307	0.15
		Precipitation	Transport	0.026	0.09
TCE	1,183	RCRA sites within 1 km of well^c^	Source	0.196	0.10
1,2,4-TMB	1,165	Agricultural land use	Source	0.004	0.05
		Precipitation	Transport	−0.046	−0.05
Toluene	1,178	Aquifer type	Transport	−0.600	−0.09
		Dissolved-oxygen content	Fate	0.657	0.11

LUST, leaking underground storage tank.

a Moran et al. (2006) provide a thorough discussion of logistic regression analysis used in this study, including the statistical approach, ancillary variables, and sources of data.

b Variables strongly associated with the occurrence of VOCs are those with absolute values of standardized coefficients of ≥0.1.

c Number of sites within a 1-km radius of well.

**Table 3:** Six regulated VOCs were detected at concentrations greater than U.S. EPA MCLs in domestic well samples, five of which are regulated carcinogens in drinking water, and most may adversely affect the liver.

Compound	VOC group	MCL (μg/L)	No. of sampled wells	No. of samples with conc > MCL	Carcinogen^a^	Potential health effects from exposure to conc > MCL^b^
DBCP	Fumigant	0.2	1,962	14	Yes	Reproductive problems; increased risk of cancer
1,1-Dichloroethene	Org syn	7	2,400	1	No	Liver problems
1,2-Dichloropropane	Fumigant	5	2,400	3	Yes	Increased risk of cancer
Ethylene dibromide	Fumigant	0.05	2,085	3	Yes	Problems with liver, stomach, reproductive system, or kidneys; increased risk of cancer
PCE	Solvent	5	2,371	5	Yes	Liver problems; increased risk of cancer
TCE	Solvent	5	2,400	6	Yes	Liver problems; increased risk of cancer

Abbreviations: conc, concentration; Org syn, organic synthesis compound.

a Considered a probable or likely human carcinogen by the U.S. EPA (2006b).

b Data from U.S. EPA (2003).
